# Mixed adeno-neuroendocrine neoplasm (MiNEN) of the small intestine presenting as sub-occlusive syndrome: a rare and challenging diagnosis

**DOI:** 10.1097/RC9.0000000000001033

**Published:** 2026-09-23

**Authors:** Mouataz Aabalou, Ahmed Jidou Vadel, Anas Derkaoui, Abdelali Guellil, Rachid Jabi, Mohammed Bouziane

**Affiliations:** a Department of General Surgery, Mohamed VI University Hospital, Oujda, Morocco; b Faculty of Medicine and Pharmacy, Laboratory of Anatomy, Microsurgery and Surgery Experimental and Medical Simulation (LAMCESM), Mohammed 1st University, Oujda, Morocco

**Keywords:** adenocarcinoma, intestinal neoplasms, MiNEN, mixed neoplasms, neuroendocrine tumors

## Abstract

**Introduction and importance:**

Mixed neuroendocrine–non-neuroendocrine neoplasms (MiNENs) are rare gastrointestinal tumors characterized by the coexistence of a neuroendocrine and a non-neuroendocrine component within the same lesion, each representing ≥30% of the tumor volume according to the 2019 WHO classification. Diagnosis remains challenging due to nonspecific symptoms, clinical variability, and limitations of imaging and biopsy sampling. MiNENs of the small intestine are particularly rare, and presentation as subocclusive syndrome is uncommon.

**Case presentation:**

We report the case of a 72-year-old patient admitted with a three-day history of subocclusive syndrome. Clinical examination revealed a diffusely tender abdomen without peritonitis, and laboratory tests were within normal limits. CT imaging showed an organic small-bowel obstruction upstream of extensive inflammatory-appearing jejunal thickening. Exploratory laparotomy revealed a stenosing jejunal mass located 40 cm from the duodenojejunal flexure, associated with multiple mesenteric lymph nodes. Segmental resection with termino-terminal anastomosis was performed. Histopathology identified a MiNEN composed of a moderately differentiated adenocarcinoma and a neuroendocrine carcinoma, classified as pT3N0Mx, with tumor-free margins.

**Clinical discussion:**

This case highlights the diagnostic difficulty with MiNENs, which are often mistaken for single-component tumors due to low biopsy sensitivity and the absence of specific radiological features. Diagnosis is most frequently established from surgical specimens. Management relies primarily on complete resection for localized disease, while adjuvant therapy is guided by the most aggressive tumor component.

**Conclusion:**

The rarity and aggressiveness of MiNENs require a multidisciplinary approach, as well as the publication of additional cases to enhance understanding of their biological behavior and to optimize therapeutic strategies.

## Introduction

Mixed neuroendocrine–non-neuroendocrine neoplasms (MiNENs) constitute a rare and heterogeneous group of gastrointestinal tumors[1], characterized by the coexistence within the same lesion of a neuroendocrine component and a non-neuroendocrine component, each accounting for at least 30% of the tumor mass according to the 2019 WHO classification[2]. Their pathophysiology remains poorly understood, as does their natural history, due to the limited number of reported cases and the wide variability of clinical presentations[3]. MiNENs of the small intestine are particularly exceptional[1] and most often present with nonspecific symptoms such as abdominal pain, anemia, or elevated inflammatory markers, making preoperative diagnosis challenging. Presentations manifesting as subocclusive or occlusive syndromes are even more uncommon. In this context, we report a case of jejunal MiNENs diagnosed in an emergency setting in a patient admitted with a subocclusive syndrome, and discuss the diagnostic, histological, and therapeutic particularities of this rare entity.

HIGHLIGHTS

- Jejunal mixed neuroendocrine–non-neuroendocrine neoplasms (MiNENs) are exceptionally rare gastrointestinal tumors.
- MiNENs may present with acute or subacute small-bowel obstruction, complicating preoperative diagnosis.
- Radiological and biological findings are often nonspecific, with a definitive diagnosis established only after surgical resection.
- Histopathological examination with immunohistochemistry is essential to identify both tumor components.
- Complete surgical resection remains the cornerstone of treatment for localized jejunal MiNENs.
- Reporting emergency presentations of small-intestinal MiNENs contributes to a better understanding of their clinical behavior and management.

This work has been reported in accordance with the SCARE Guidelines 2025[4].

## Case presentation

The patient was a 72-year-old man with a history of transurethral bladder resection performed 2 years earlier for a suspected bladder tumor, for which histopathological examination was negative. He presented to the emergency department with a 3-day history of subocclusive syndrome characterized by cessation of stool passage while still passing gas, associated with generalized abdominal pain and no additional symptoms (no vomiting or gastrointestinal bleeding), occurring in an afebrile context.

Clinical examination found a conscious patient who was hemodynamically stable, respiratory stable, and afebrile. Abdominal examination revealed diffuse abdominal tenderness without abdominal distension; hernial orifices were free. Digital rectal examination revealed traces of stool.

Laboratory investigations showed a normal complete blood count (WBC 8000/mm^3^, Hb 15 g/dL), normal CRP (2 mg/L), normal albumin level (44 g/L), and elevated serum creatinine (12 mg/L). Electrolytes and the remainder of the biological tests were normal. Tumor markers were negative (CA19-9 = 21 U/mL, CEA < 1.7 ng/mL). Fecal calprotectin and tuberculosis workup were negative.

A contrast-enhanced abdominopelvic CT scan showed a mechanical small bowel obstruction upstream of an extensive, inflammation-like thickening of the jejunal loops, with no signs of intestinal ischemia (Fig. 1).

**Figure 1.**
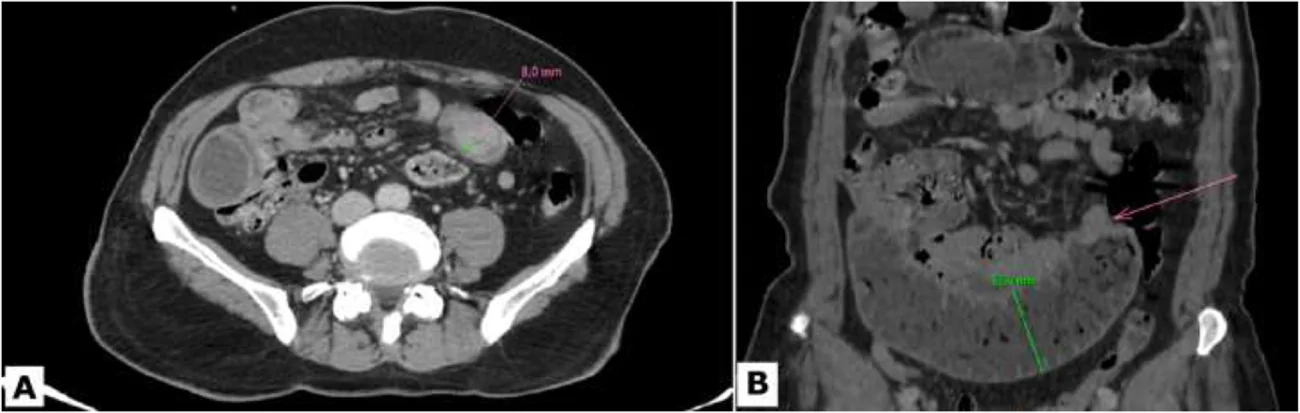
Axial (A) and coronal (B) views of an abdominal CT scan show small-bowel dilatation measuring 52 mm upstream of a wall thickening of approximately 8 mm (purple arrow).

The patient underwent an exploratory laparotomy, which revealed a stenosing small-bowel mass located 40 cm from the first jejunal loop, with upstream small-bowel distension. There was no invasion of adjacent structures. Multiple lymph nodes were noted in the mesentery facing the mass, extending along the proximal jejunal arterial arcades.

A segmental small bowel resection was performed, with proximal and distal resection margins greater than 5 cm, followed by a manual termino-terminal jejunojejunal anastomosis (Fig. 2).Histopathological examination of the resected jejunal specimen revealed a mixed adeno-neuroendocrine carcinoma composed of a moderately differentiated adenocarcinoma and a neuroendocrine carcinoma that extended into the subserosa and was classified as pT3N0Mx, with clear resection margins (Fig. 3).

**Figure 2.**
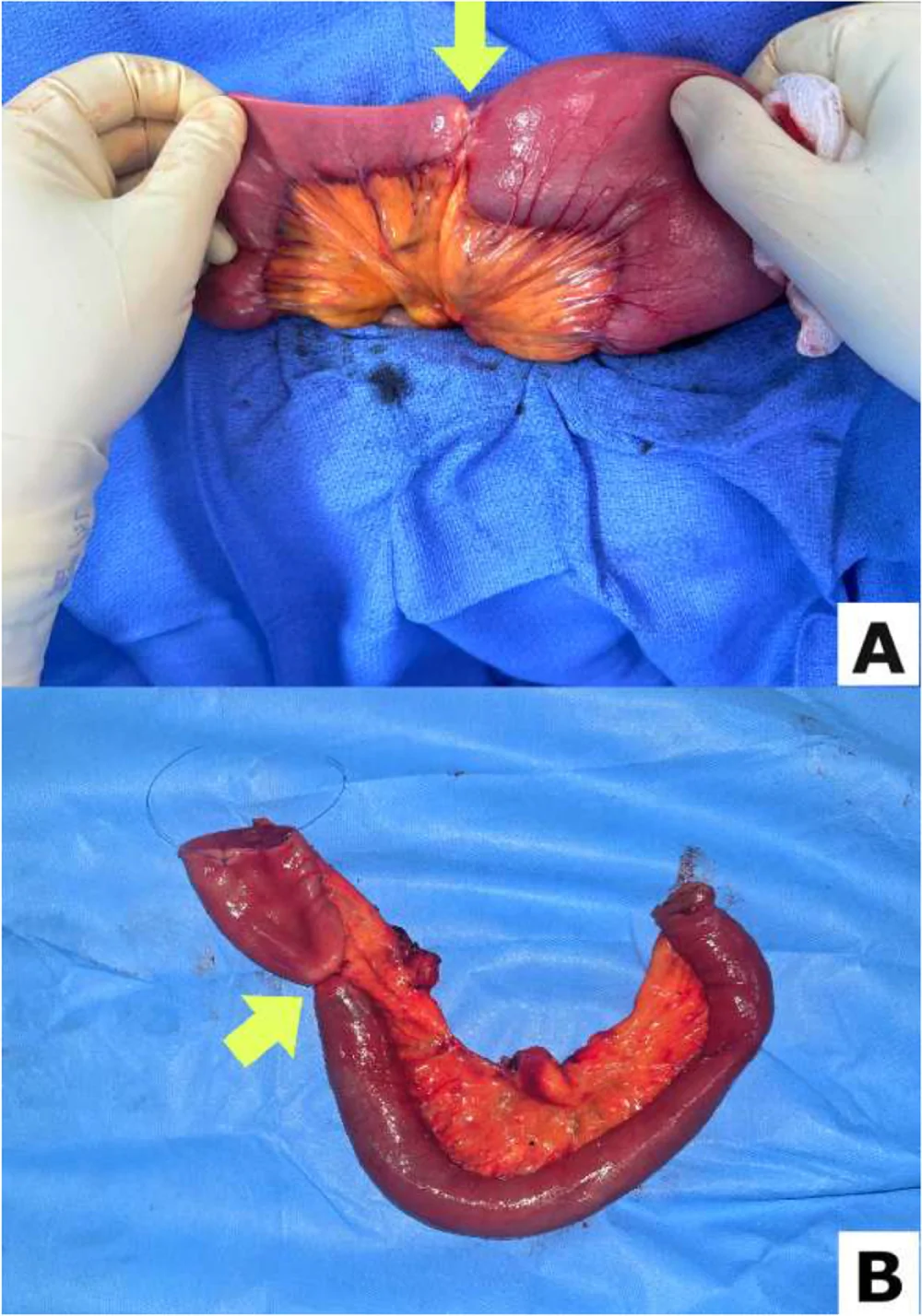
(A) Intraoperative image showing a stenosing mass (arrow) with a dilated proximal small-bowel loop. (B) An image of the surgical specimen after resection, showing the stenosing mass (arrow).

**Figure 3.**
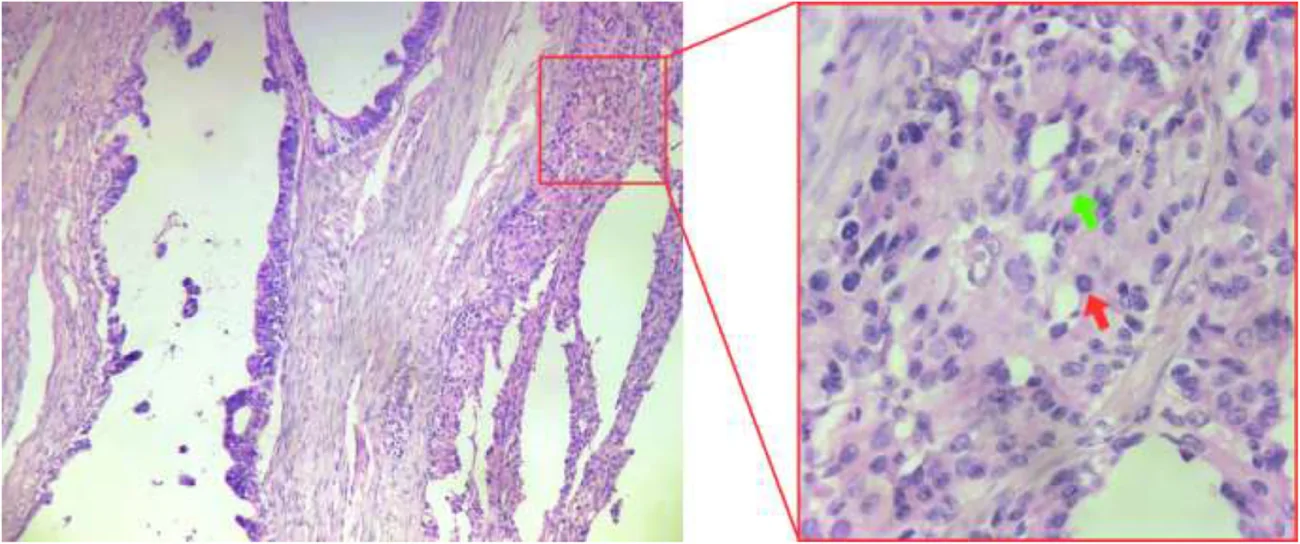
Histopathological photomicrograph (H&E-stained) showing a biphasic proliferation composed of two components: an adenocarcinomatous component with glandular architecture, consisting of columnar cells with enlarged, hyperchromatic nuclei and abundant eosinophilic cytoplasm, and a second neuroendocrine component arranged in small nests of relatively uniform cells. The boxed area, shown at higher magnification (right), illustrates cells displaying a characteristic “salt-and-pepper” chromatin pattern typical of neuroendocrine tumors (green arrow) and a mitotic figure (red arrow).

The neuroendocrine component showed diffuse positivity for synaptophysin and chromogranin A, with focal CD56 expression and a Ki-67 proliferation index of approximately 45%, consistent with a high-grade neuroendocrine carcinoma (G3) according to the 2019 WHO classification. The adenocarcinoma component was positive for CDX2 and CK20, consistent with intestinal-type differentiation, and negative for CK7. Mismatch repair (MMR) immunohistochemistry showed retained expression of MLH1, MSH2, MSH6, and PMS2, indicating microsatellite-stable (MSS) disease. Each component accounted for at least 30% of the total tumor volume, fulfilling the 2019 WHO diagnostic criteria for MiNEN. These findings were reviewed and validated by an experienced gastrointestinal pathologist.

The postoperative course was uneventful, and the patient was discharged on the sixth postoperative day. He was subsequently referred to the medical oncology department for adjuvant chemotherapy.

Following surgical resection, the case was presented at our institution’s multidisciplinary tumor board (MTB), which convened specialists in digestive surgery, medical oncology, gastroenterology, and pathology. In view of the pathological findings – pT3N0Mx MiNEN with a high-grade neuroendocrine carcinoma component (Ki-67 approximately 45%), R0 resection margins, and microsatellite-stable status – the MTB recommended adjuvant systemic chemotherapy. Given the aggressiveness of the neuroendocrine carcinoma component and in accordance with current recommendations advocating that treatment be guided by the most aggressive tumor element, a platinum-based regimen combined with etoposide was proposed as the first-line adjuvant protocol. The patient was referred to the medical oncology department and accepted the proposed therapeutic plan following an informed discussion.

## Discussion

MiNENs, formerly referred to as mixed adenoneuroendocrine carcinoma (MANEC), are rare entities defined by the WHO 2019 classification as tumors composed of both a neuroendocrine and a non-neuroendocrine component, each representing at least 30% of the tumor volume[2]. MiNENs include all possible combinations, such as adenocarcinoma and neuroendocrine carcinoma, squamous cell carcinoma and neuroendocrine carcinoma, and others. The former terminology, MANEC, only referred to the combination of adenocarcinoma and neuroendocrine carcinoma; therefore, recent literature no longer recommends its use, as it is obsolete and too restrictive[1].

The current definition of MiNENs allows these tumors to be distinguished from tumors showing only focal neuroendocrine differentiation. However, the ≥30% cutoff for each component, although widely adopted, remains controversial because even a small aggressive component may influence prognosis, and this threshold limits the ability to diagnose MiNENs in biopsy specimens[5].

Though the pathophysiology of MiNENs is poorly understood, it is suggested that MiNENs derive from a monoclonal precursor, which later acquires bi-phenotypic features through the carcinogenic process[6].

MiNENs are rarely revealed by intestinal obstruction, whether partial or complete. Most reported cases are associated with a history of chronic inflammatory bowel disease or atypical colonic masses, and symptoms are often nonspecific – abdominal pain, gastrointestinal bleeding, or general deterioration – leading to delayed diagnosis^[^5,7^]^.

Imaging and biopsy are not always reliable for detecting mixed differentiation. Radiologically, MiNENs have no specific features, and CT, MRI, or even PET scans may underestimate tumor heterogeneity[8]. In non-emergency settings, the complementary use of Ga-68 DOTATATE PET/CT and FDG-PET/CT is increasingly recognized to better capture the biological heterogeneity of MiNENs, given that the neuroendocrine and non-neuroendocrine components may differ substantially in somatostatin receptor expression and metabolic activity; however, in the present case, the acute sub-occlusive presentation required urgent surgical intervention, rendering such functional imaging workup infeasible. Likewise, limited biopsy samples have low diagnostic accuracy, as they may fail to capture both components, resulting in frequent underdiagnosis^[^3,7^]^. This highlights the importance of a comprehensive pathological assessment, including morphological and immunohistochemical evaluation (neuroendocrine markers: synaptophysin, chromogranin, CD56, and Ki-67), ideally performed by an experienced pathologist, to establish the diagnosis and determine tumor grade[9].

Therapeutic management of MiNENs remains challenging due to their rarity. No standardized treatment guidelines have been clearly established, and recommendations rely mainly on small case series. For localized, resectable tumors – such as in our patient – complete surgical resection (R0) remains the gold standard and provides the best survival outcomes. Adjuvant chemotherapy is often considered, especially in the presence of a high-grade neuroendocrine component or lymph node involvement. In cases of locally advanced or metastatic disease, systemic chemotherapy is indicated, usually guided by the most aggressive component (often high-grade neuroendocrine carcinoma; platinum–etoposide regimens)^[^9,10^]^. In our patient, the emergency presentation with sub-occlusive syndrome required urgent surgical intervention, which allowed both definitive histological diagnosis and curative resection. In elderly patients, adjuvant chemotherapy decisions should integrate performance status, comorbidity burden, and organ function alongside tumor biology, as standard platinum-etoposide regimens carry significant hematological and renal toxicity requiring individualized risk-benefit assessment.

The prognosis of MiNENs is generally poor, largely due to the aggressiveness of the neuroendocrine component, which is often high grade^[^11,12^]^. This underscores the importance of reporting additional cases to enhance understanding of their biological behavior and to support the development of individualized, multidisciplinary management strategies, while awaiting specific recommendations based on larger cohorts.

Small-intestinal MiNENs are exceptionally rare, with fewer than 50 cases documented in the indexed literature as of 2024; the majority involve the ileum rather than the jejunum, as in our patient. Obstruction as a presenting feature has been reported in only a minority of cases; most patients present with chronic abdominal pain, gastrointestinal bleeding, or weight loss, often after a prolonged diagnostic delay. Preoperative diagnosis was achieved in fewer than 10% of published cases, because biopsy sampling typically identifies only one component – most often the adenocarcinoma – consistent with our experience, in which the mixed nature of the tumor was revealed only on the surgical specimen. The European multicenter retrospective study by Frizziero *et al*[7] and the systematic review by Frizziero *et al*[3] report a consistently poor prognosis, particularly when a high-grade neuroendocrine carcinoma component is present, with median overall survival ranging from 8 to 22 months in patients with advanced disease. Localized, resected cases carry a relatively more favorable prognosis, though long-term data remain sparse.

## Conclusion

MiNENs consistently present a diagnostic challenge due to the non-specific nature of symptoms, the limitations of imaging, and the low sensitivity of biopsy sampling, with diagnosis often established only after surgical resection. Reported cases show that most patients are diagnosed at an advanced stage, frequently with metastases, owing to the aggressiveness of the neuroendocrine component. Complete (R0) surgical resection remains the cornerstone of treatment for localized disease, whereas adjuvant therapy should be individualized according to the most aggressive component. The rarity of MiNENs justifies the publication of additional cases to improve understanding of their biological behavior and to support the development of future therapeutic recommendations.

## Data Availability

Not applicable.
